# Effect of Iron Source and Medium pH on Growth and Development of *Sorbus commixta* In Vitro

**DOI:** 10.3390/ijms22010133

**Published:** 2020-12-24

**Authors:** Jie Xiao, Yoo Gyeong Park, Ge Guo, Byoung Ryong Jeong

**Affiliations:** 1 Department of Horticulture, Division of Applied Life Science (BK21 Program), Graduate School of Gyeongsang National University, Jinju 52828, Korea; xiaojsicau@163.com (J.X.); rainbowmaomao317@gmail.com (G.G.); 2 Institute of Agriculture and Life Science, Gyeongsang National University, Jinju 52828, Korea; iuyiuy09@naver.com; 3 Research Institute of Life Science, Gyeongsang National University, Jinju 52828, Korea

**Keywords:** antioxidant enzyme, chlorophyll, chlorosis, ferric chelate reductase, iron

## Abstract

*Sorbus commixta* is a valuable hardwood plant with a high economical value for its medicinal and ornamental qualities. The aim of this work was to investigate the effects of the iron (Fe) source and medium pH on the growth and development of *S. commixta* in vitro. The Fe sources used, including non-chelated iron sulfate (FeSO_4_), iron ethylenediaminetetraacetic acid (Fe-EDTA), and iron diethylenetriaminepentaacetic acid (Fe-DTPA), were supplemented to the Multipurpose medium with a final Fe concentration of 2.78 mg·L^−1^. The medium without any supplementary Fe was used as the control. The pH of the agar-solidified medium was adjusted to either 4.70, 5.70, or 6.70. The experiment was conducted in a culture room for six weeks with 25 °C day and night temperatures, and a 16-h photoperiod with a light intensity of 50 mmol·m^−2^·s^−1^ photosynthetic photon flux density (PPFD). Both the Fe source and pH affected the growth and development of the micropropagated plants in vitro. The leaves were greener in the pH 4.70 and 5.70 treatments. The tissue Fe content decreased with the increase of the medium pH. The leaf chlorophyll content was similar between plants treated with FeSO_4_ and those with Fe-EDTA. The numbers of the shoots and roots of plantlets treated with FeSO_4_ were 2.5 and 2 times greater than those of the control, respectively. The fresh and dry weights of the shoot and the root were the greatest for plants treated with Fe-EDTA combined with pH 5.70. The calcium, magnesium, and manganese contents in the plantlets increased in the pH 5.70 treatments regardless of the Fe source. Supplementary Fe decreased the activity of ferric chelate reductase. Overall, although the plantlets absorbed more Fe at pH 4.70, Fe-EDTA combined with pH 5.70 was found to be the best for the growth and development of *S. commixta* in vitro.

## 1. Introduction

*Sorbus* (*Rosaceae*) is a genus comprised of about 100–200 species of trees and shrubs that have been variously utilized in ornamental, industrial, edible, and medical applications [1]. *S. commixta*, also known as Japanese rowan is an ornamental tree distributed in China, Japan, Korea, and Russia [2]. This plant has been reported to show many pharmacological effects, such as anti-oxidative, anti-ice-nucleated, anti-vascular-inflammatory, anti-lipid-peroxidative, anti-atherogenic, and vasorelaxant [3]. Its stem bark has been used to treat cough, asthma, bronchial disorders, gastritis, and dropsy [4]. *S. commixta is* conventionally propagated by seeds, but germination of fresh matured seeds is hindered by dormancy and inhibitors of pulp [5]. Moreover, seed propagation leads to a high variability. Micropropagation is an important method for maintaining unique characteristics, as well as overcoming the problematically low cutting yield. And the in vitro culture environment is more controlled than the in vivo environment, and by maintaining unique environmental characteristics, we can apply the experimental results directly to production without other environmental constraints, we are still working on finding the best conditions for the in vitro cultures of this species.

Iron (Fe) is the fourth most abundant element in the earth’s crust, at 5%, and the soil contains from 1 to 5% (on average 3.2%) Fe. On the other hand, the Fe content in plants is normally only 0.005% [6,7]. Fe is an important elemental nutrient for plants; it not only participates in the synthesis of organelles, such as the chloroplast, mitochondria, and palisade tissues, but also acts as a cofactor for numerous enzymatic processes [8]. Lack of Fe causes decreased contents of chlorophylls, the most obvious symptom is stunted growth and induction of chlorosis in the youngest leaves. One of the main causes of such Fe deficiency is the low solubility and dissolution rate of the solid phase Fe in soils [9]. Plants have thus accordingly developed a mechanism to facilitate the absorption of Fe in their roots. Generally, for dicotyledons and non-graminaceous plants, the rate of Fe-III reduction and corresponding splitting of Fe-III-chelates at the plasma membrane are enhanced by increasing the activity of plasma membrane-bound reductase. Further, roots produce ferric chelate reductase (FCR) to catalyze Fe^3+^ to Fe^2+^, then the Fe uptake is facilitated by the high affinity transporter IRT1 [10], this mechanism is called Strategy Ι. *Gramineae* utilize a different strategy (Strategy ΙΙ), that’s based on chelation, and the release of phytosiderophores (non-proteinogenic amino acids) with a high affinity for ferric ions, which mobilize the sparingly soluble inorganic ferric ionic compounds [11]. Therefore, the application of chelated Fe can reduce the degree of leaf chlorosis and significantly increase the growth in calcareous soil [12].

For acidophilous plants, however, only supplementing chelated Fe is not a good way to treat chlorosis because the plants have a reduced uptake of a single element, especial Fe, under a high pH. An increase in the growing medium pH means an increase in the concentration of carbonate (CO_3_^2−^) and bicarbonate (HCO_3_^−^), and the solubility of Fe is reduced due to the consumption of H^+^ by HCO_3_^−^ [13]. Local acidification of calcareous soils in quince orchards can alleviate Fe chlorosis and significantly increase the contents of chlorophyll and Fe^2+^ in quince [14]. Different species respond differently to the inhibition of Fe absorption and transport, and therefore exhibit different sensitivity and tolerance. *S. aucuparia* was chlorosis-susceptible at pH 7.2, while *Plantago media* and *Potentilla crantzii* were chlorosis-resistant at the same pH [15]. Supplementing Fe and adjusting the pH are effective means of alleviating chlorosis, compared to either one of them alone [16]. A study was made, therefore, to investigate the responses of *S. commixta* to different supplementary Fe sources and pH levels and explore appropriate Fe source and pH for micropropagation of *S. commixta* which often exhibited chlorosis.

## 2. Results

### 2.1. Morphology and Growth Parameters Analyses

**Figure 1:**
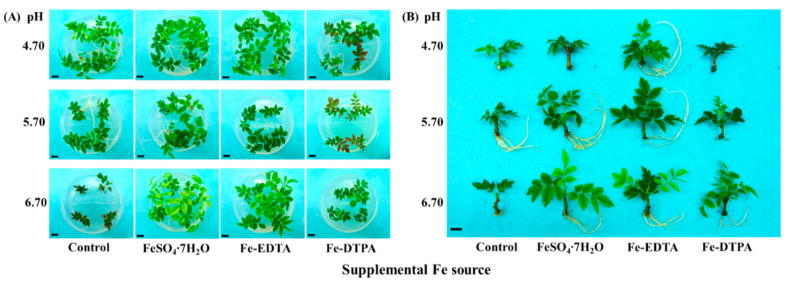
Photographs showing morphology of *S. commixta* as affected by the Fe source and medium pH after 6 weeks of culture: (**A**), a top view of the plantlets still in the medium; and (**B**), representative individual plantlets. The bars in each photograph indicate 1 cm.

It has been clearly observed that *S. commixta* (Figure 1A,B) exhibits increased growth upon receiving Fe supplementation. The roots were developed after 6 weeks of culture, except in the Fe-DTPA treatment at pH 4.70 and in the control at pH 6.70 (Figure 1B and Table 1). The number of roots, fresh and dry root weights, and leaf area were the greatest in the Fe-EDTA treatment at pH 5.70, and the number of roots were higher than that in the control. Moreover, the treatment with FeSO_4_ at pH 5.70 had the greatest number of leaves, fresh and dry shoot weights, measurements which were 3.5, 5.4, and 3 times higher than the corresponding measurements in the control. However, the relative water content and water content were not significantly different among the treatments.

The color measurement values made on the *S. commixta* leaves are shown in Table 2. Fe supplemental and pH treatments significantly affected the color of leaves. The lightness (L*), red (+)/green (−) color attribute (a*), and yellow (+)/blue (−) color attribute (b*) values respectively represent the lightness, red-to-green, and blue-to-yellow. The a* values (−31 and −25) of the leaves in the pH 4.70 treatments with supplementary FeSO_4_ or Fe-EDTA show that these were greener than others. In the control at pH 6.70 and in the Fe-DTPA treatment at pH 4.70 and 5.70, the leaf color had higher a* values, and were nearly red. Moreover, the b* values (60, 52, and 46) of the leaves show that they were yellower in the pH 6.70 treatments with supplementary Fe, which meant that the yellow color ratio increased with the increase in the pH (Figure 1A,B).

### 2.2. Chlorophyll Contents, Soluble Proteins, and Antioxidant Enzyme Activities

**Figure 2:**
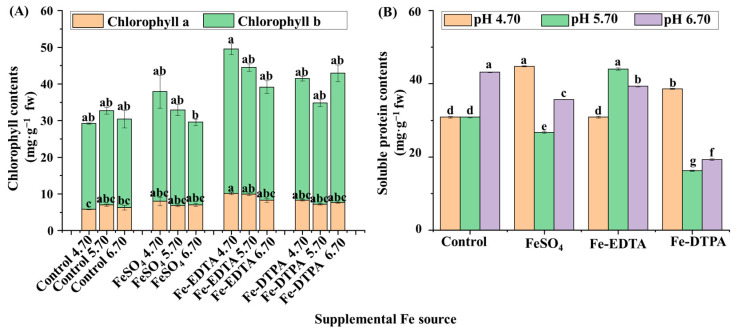
The contents of chlorophyll (**A**) and soluble proteins (**B**) in *S. commixta* after 6 weeks of culture; fw, fresh weight. Lowercase letters indicate significant differences by the Duncan’s multiple range test at *p* ≤ 0.05.

**Figure 3:**
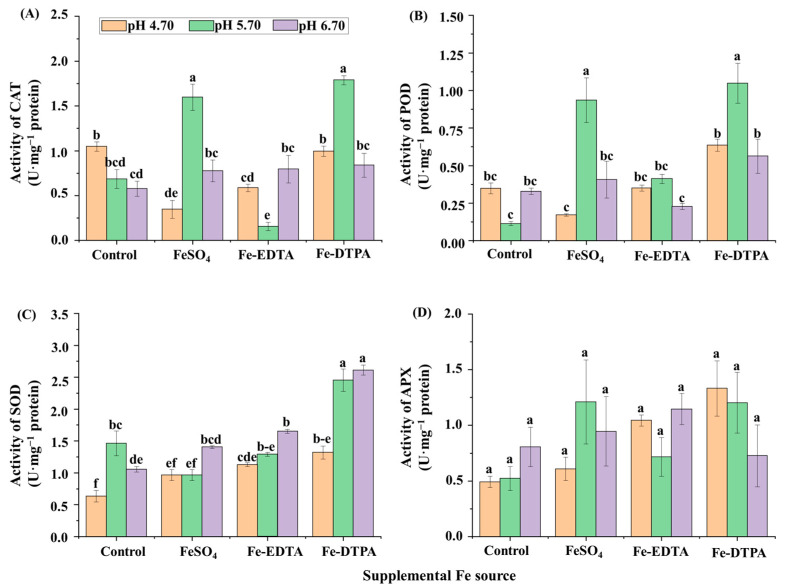
The activities of CAT (**A**), POD (**B**), SOD (**C**), and APX (**D**) in *S. commixta* after 6 weeks of culture. Lowercase letters indicate significant differences by the Duncan’s multiple range test at *p* ≤ 0.05.

The chlorophyll contents decreased with the increase of the medium pH in treatments with FeSO_4_ and Fe-EDTA, and the highest chlorophyll a and b contents were found in the treatment with Fe-EDTA at pH 4.70 (Figure 2A). Higher soluble protein contents were observed in plants grown in the control at pH 6.70, with FeSO_4_ at pH 4.70, and with Fe-DTPA at pH 5.70, and these values were 2.5 times that with Fe-DTPA at pH 5.70 (Figure 2B). The CAT activity was greater in the treatments at pH 5.70 with FeSO_4_ and Fe-DTPA, which were respectively 9.6 and 10.8 times than that in the control at pH 5.70 (Figure 3A). Similarly, the POD activity was greater in the treatments at pH 5.70 with FeSO_4_ and Fe-DTPA, which were respectively 7.4 and 8.4 times higher than that in the control at pH 5.70 (Figure 3B). The SOD activity increased with the increase in the medium pH regardless of the Fe source, and the SOD activity in plants grown with Fe-DTPA at pH 6.70 was 4 times than that of the control at pH 4.70 (Figure 3C). There were no significant differences in the APX activity among treatments with different supplementary Fe sources (Figure 3D).

### 2.3. FCR Activities

**Figure 4:**
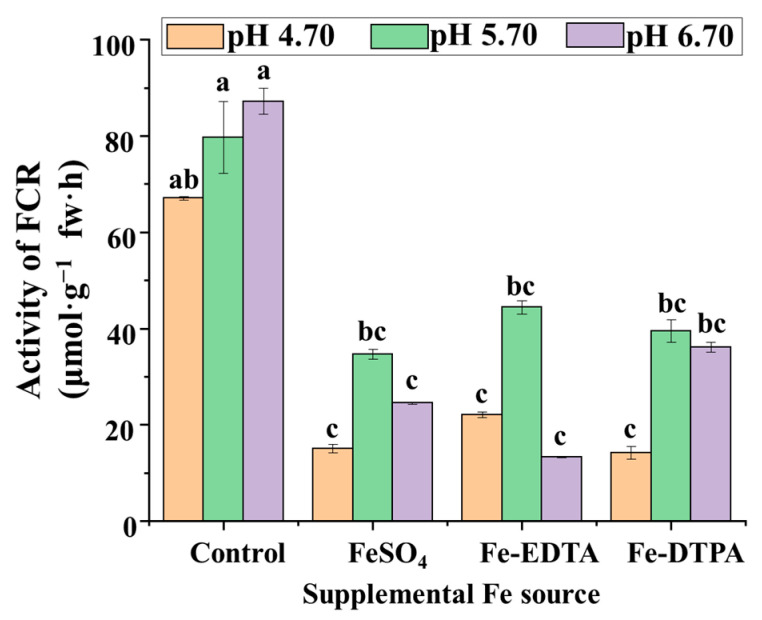
The FCR activities in *S. commixta* after 6 weeks of culture; fw, fresh weight. Lowercase letters indicate significant differences by the Duncan’s multiple range test at *p* ≤ 0.05.

The results of this study showed that FCR activities in the control group were 2 to 4 times that in treatments supplemented with different Fe sources, which indicates that FCR activities decrease with Fe supplementation (Figure 4). Among the treatments with FeSO_4_, Fe-EDTA, and Fe-DTPA, the highest FCR activities were observed at pH 5.70. FCR activities in the treatment with Fe-DTPA at pH 6.70 resulted in the second highest FCR activities among the treatments. The FCR activities were similar among treatments with FeSO_4_ at pH 4.70 and 6.70, with Fe-EDTA at pH 4.70 and 6.70, and with Fe-DTPA at pH 4.70.

### 2.4. Analysis of Stomata

**Figure 5:**
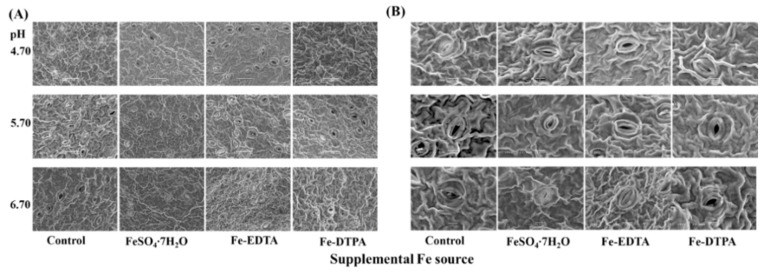
The SEM images of leaf stomata of *S. commixta* after 6 weeks of culture. Bars indicate 50 μm (**A**) and 10 μm (**B**).

**Figure 6:**
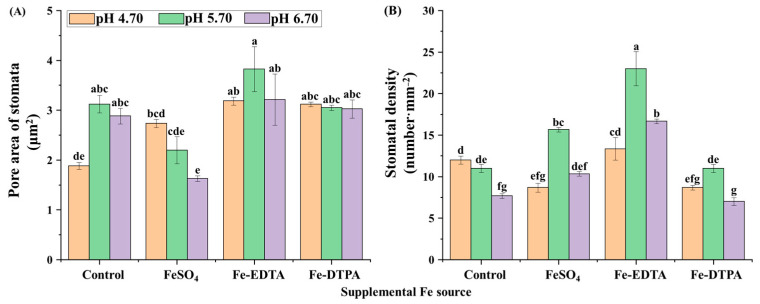
Pore area of the stomata (**A**) and the stomatal density (**B**) in leaves of *S. commixta* after 6 weeks of culture. Lowercase letters indicate significant differences by the Duncan’s multiple range test at *p* ≤ 0.05.

Proper development and compactness of stomata were observed, as shown in Figure 5. Opening border of guard cells is raised and the stomata resemble reniform or lip structures. The pore area of the stomata and stomatal density are shown in Figure 6. The greatest single pore area was observed in the treatment with Fe-EDTA at pH 5.70, while the smallest single pore area was observed in the control at pH 4.70. In treatments with supplementary FeSO_4_ and Fe-DTPA, the stomatal area decreased with the pH, while no significant differences were observed in the stomatal size in the treatment with Fe-EDTA with variations in the pH. In the microscopical field, treatments led to the greatest number of stomata at pH 5.70, regardless of the Fe source.

### 2.5. Macro- and Micro-Nutrients Content

Treatments with supplementary Fe significantly affected the content of macro- and micro-nutrients in *S. commixta* (Table 3). The tissue Fe content decreased with the increase in the medium pH, while the cupper (Cu) content decreased with decreasing Fe content. With no supplementary Fe, plants in the control had lower Fe contents, especially at pH 6.70. The control at pH 6.70 also had the lowest contents of pottassium (K), manganese (Mn), Fe, and sulfur (S). Contents of calcium (Ca), zinc (Zn), Mn, boron (B), and S in the plantlets increased in the pH 5.70 treatments regardless of the Fe source. The contents of K, Ca, magnesium (Mg), phosphorus (P), and S were higher in the treatment with Fe-EDTA at pH 5.70. Similarly, the contents of Zn, Mn, B, and silicon (Si) were higher in the treatment with FeSO_4_ at pH 5.70. The contents of K, Ca, Mg, Zn, B, and Si were lower in the treatment with Fe-DTPA at pH 4.70.

## 3. Discussion

Fe application is particularly important in micropropagation. Boamponsem [17] found that the fresh weight and area of callus growth decreased with an absence of Fe in the growth medium in potato, and the texture of the calli was slightly soft and brittle. Similarly, in micropropagation of *Vitis*, Fe-deficiency in the medium greatly affected the fresh weight of calli regardless of the genotype of Fe efficiency. Moreover, as the Fe applied to the leaves is transported from the stomata to the phloem, it will be absorbed by the parenchyma cells surrounding the substomatal chambers [18], which is limited by the rate of transpiration and the top is not a priority supply. Therefore, the most obvious symptom of associated Fe deficiency is stunted growth and induction of chlorosis in the youngest leaves. During the regeneration of the axillary shoots of *Nicotiana benthamiana* over 4 weeks, numerous plants showed leaf chlorosis associated with Fe deficiency. After these plants were transferred to the MS medium containing double strength Na_2_FeEDTA, they quickly recovered from chlorosis and turned healthily green [19]. The results of the current study exhibited strongly enhanced *S. commixta* growth with supplementary FeSO_4_, Fe-EDTA, and Fe-DTPA in the multipurpose medium. The supplemental Fe enhanced the number of leaves and root formation after 6 weeks of culture. In addition, the solubility of the Fe sources varies in different pHs because Fe chelates differ in their ability to withstand displacement of their metal. When the pH is lower than 5.5, FeSO_4_ has a high solubility, which quickly decreases with increasing pH [20,21]. At high pH, however, Fe-DTPA has higher soluble Fe than Fe-EDTA did [22]. In this study, the promotion of plant growth by supplemental Fe was affected by the Fe source and pH. All the observations indicated that the utilization of supplemental FeSO_4_ and Fe-EDTA with a low pH, and supplemental Fe-DTPA at pH 6.70, could promote the growth and development of *S. commixta* in vitro, and decrease chlorosis.

The leaf is a main sink of Fe and 80% of the metal in the leaf is located in the chloroplasts. The material composition of the leaves is affected by these metal ions. Protoporphyrin ΙΧ the intermediate product of chlorophyll synthesis, which combines with Mg into Mg-protoporphyrin, and then monovinyl protochlorophyllide a can be synthesized, further producing chlorophyll a. On the other hand, protoporphyrin ΙΧ combined with Fe produces ferroheme. Kobayashi [23] found Mg deficiency caused Fe-responsive gene increase, and it seems that Fe has more opportunities to synthesize ferritin under the condition of Mg shortage. Moreover, ferritin is involved in thylakoid synthesis and photosynthesis [24]. Thus Fe-deficiency will cause thylakoid growth to stagnate, and decrease the densities of chlorophyll a and b, P700, and cytochrome f [25]. It was reported that the proportion of xanthophyll to chlorophyll contents in leaves of potato increased with Fe-deficiency [26]. Similarly, with Fe-deficiency *Beta vulgaris* leaves lost 95% of the chlorophyll, *β*-carotene, and neoxanthin, while the xanthophyll content was less affected [27]. Supplemental Fe stimulated the accumulation of chlorophyll [28]. Therefore, the decrease in the chlorophyll content is the main reason of chlorosis caused by Fe-deficiency and the utilization of Fe can be clearly reflected by content of chlorophyll. In this study, the trend of the chlorophyll content and that of the Fe content are the same, where the treatment with Fe-EDTA at pH 4.70 resulted in the highest chlorophyll content and simultaneously the greenest leaves. It was observed that supplemental Fe-EDTA at pH 4.70 increased the chlorophyll content and accordingly decreased chlorosis, which was in agreement with the findings of Nemati Lafmejani [29] and Mann [30]: a significant increase in the chlorophyll content with foliar application of Nano-Fe and FeSO_4_. It was worth noting that the leaves of plantlets grown in the control at pH 6.70, with Fe-DTPA at pH 4.70 and pH 5.70 displayed varying degrees of color change: leaves turned brown when grown in the control at pH 6.70, while the leaves grown with supplementary Fe-DTPA at pH 4.70 and pH 5.70 were metallically luster, and the degree of bronzing decreased with the increase of the pH when Fe-DTPA was supplemented. It is speculated that this is related to the Fe content, where a significant increase in the Fe in all treatments that supplemented Fe, especially the treatments with Fe-DTPA at pH 4.70, 5.70, and 6.70, where the Fe content was respectively 11, 8, and 6 times than that in the control. It is reported that the critically toxic Fe content of rice is 0.3 mg·g^−1^ shoot dry weight, and that of *Tagetes erecta* L. is 1.0 mg·g^−^^1^ [31,32], while the critically toxic Fe content for *S. commixta* is still unclear. It depends on the developmental/growth stage, physiological status, and variety of plant, which are all involved with the nutrient contents. For instance, Zn and Mn contents are negatively affected by a high Fe concentration [33]. In this study, the Zn and Mn contents decreased with the Fe content when the Fe concentration was greater than 3.0 mg·g^−1^ dry weight, while the Ca, Mg, B, and P contents were the lowest when the Fe concentration was the greatest. It is speculated that the absorption of each nutrient element is in a relatively balanced state within a certain concentration range of Fe. Supplementing Fe or other micronutrients can increase the chlorophyll content in plants. However, since a narrow range of Fe concentration divides phytotoxicity and Fe toxicity [30], there is also a risk of toxicity. Therefore, the concentration of Fe application at different pH levels needs far more research. In plants, protein signaling is more complex due to the source-sink interaction and the intimate interaction of web-like signaling networks governed by plant hormones, nutrients, and environment condition. Generally, plants produce reactive oxygen intermediates (ROS) from metabolic processes such as photosynthesis and respiration. This ROS attack on lipids, proteins, and nucleic acids causes lipid peroxidation, protein denaturation, and DNA mutation [34]. The SOD is the first line of defense against oxidation in plants, which produces H_2_O_2_ and O_2_, and then APX, CAT, and POD catalyze H_2_O_2_ to H_2_O [35]. In this study, SOD activities were not significantly different between plants grown with FeSO_4_ and with Fe-EDTA. However, SOD responded to a high Fe content and its activities significantly increased in the treatment with Fe-DTPA at pH 5.70 and 6.70. It is worth noting that the SOD activities increased in the treatment with Fe-DTPA at pH 4.70, and it is speculated that the decreased Mn and Zn contents led to the metal prosthetic (Mn-SOD and Cu/Zn-SOD) isoforms of SOD to be reduced [36]. On the other hand, ROS exceeds the range of SOD due to excessive Fe, thus the activity of SOD was inhibited. H_2_O_2_ can be reduced by CAT, POD, and APX. These three antioxidant enzymes belong to hemoproteins and their activities are affected by the Fe content [37]. Boamponsem [17] and Molassiotis [38] reported that POD and APX activities were gradually increased with a rise in the supplementary Fe levels in potato calli, and the CAT activities were suppressed in the Fe-deprived tissues of different peach rootstocks. It was illustrated that Fe is extremely important in the enzyme system. In addition, we found that the activities of CAT and POD increased at pH 5.70 regardless of the Fe source. It is interesting to note that although all three treatments, FeSO_4_ at pH 4.70, Fe-EDTA at pH 5.70, and Fe-DTPA at pH 4.70, had higher contents of soluble proteins, but lower activities of antioxidant enzymes. The treatments with FeSO_4_ and Fe-EDTA at pH 5.70 have lower contents of soluble proteins and higher activities of antioxidant enzymes. It is speculated that proteins damaged by ROS increase response of antioxidant enzymes. In vitro plants are under slight stress, and soluble protein and antioxidant enzymes are important indicators of plant metabolism and reactive oxygen species balance. The condition of plant can be clearly reflected by values of these parameters. In brief, it is beneficial for plants to maintain a ROS balance and the best medium pH to do so was pH 5.70.

The redox reaction of Fe^2+^ (ferrous) and Fe^3+^ (ferric) ions is reversible, and it can change the redox potential according to different ligand environments [39]. FCR maintains Fe homeostasis by regulating this pathway in plants, and FCR activity can be used as a criterion when screening tolerance to Fe-deficiency and Fe-toxicity of rootstocks or cultivars. In this study, the FCR activities increased in treatments with supplementary Fe at pH 5.70. FCR activities have been described to be higher at pH values below 6.5 [34]. It is worth noting that FCR activities was markedly increased in the case of Fe-deficiency, while in Fe supplemental conditions, there was a significantly negative correlation between the Fe content and the FCR activities in *S. commixta* regardless of the Fe sources, findings which are consistent with those of YiChieh Chang [40] and SoRa Lee [41]. The high FCR activities can be regarded as a reaction mechanism that absorbs a large amount of Fe by Fe-deficient plants [42], which is also applicable to *S. commixta* investigated in this study.

Stomata is a basic plant-specific organelle for transpiration and respiration, and Fe significantly affected the stomatal size and upper epidermis thickness of leaves [43,44]. Fe-deficiency caused a smaller stomatal size on the leaf surface, decreased the degree of stomatal opening, and affected the gas exchange of plants [45]. In this study, stomatal density increased with supplementary Fe treatments regardless of the Fe source in pH 5.70. It indicates that Fe and pH influence stomatal density simultaneously. However, supplementary Fe had no significant effects on the stomatal area. It is speculated that the plant may store enough Fe in the stem before transplanting to support the development of stomata.

## 4. Materials and Methods

### 4.1. Plant Materials and Culture Conditions

Actively growing shoots were collected from glasshouse-grown plants that are 1–2 years old. The shoots were washed under running tap water for 15 min, rinsed thoroughly with distilled water, and subsequently disinfected in a 70% (*v*/*v*) ethanol solution for 60 s, a 1.5% (*v*/*v*) sodium hypochlorite solution with a drop of Tween 20 for 10 min, and a 0.01% (*v*/*v*) mercuric chloride for 15 min. Each shoot was then washed 4 times with sterile distilled water. Nodal explants (1.0–1.5 cm) were placed in a culture container (113 mm × 89 mm, Phyto Technology Laboratories, Shawnee Mission, KS, USA) containing 50 mL of the MS medium (Murashige and Skoog 1962) [46], supplemented with 2.0 mg·L^−1^ BA, 0.5 mg·L^−1^ NAA, 3% (*w*/*v*) sucrose, and 0.80% (*w*/*v*) agar for shoot induction in October 2015. After 35 days, roots were induced on the half-strength MS medium supplemented with 0.5 mg·L^−1^ IBA for 30 days. Thereafter, the plantlets in vitro were subcultured on the MS medium without supplementation of any PGR [2]. The plants were subcultured every three months, and number of passages was 17 before the plantlets were used as the explant source for this research. All cultures were maintained in a growth chamber at 25 °C and with a 16-h photoperiod provided by cool white fluorescent light (40 W tubes, Philips) at an intensity of 50 mmol·m^−2^·s^−1^ photosynthetic photon flux density (PPFD).

### 4.2. Iron and pH Treatments

The regenerated shoots that were longer than 2.0 cm were excised from multiple shoots and cultured on a medium called a multipurpose medium with 3.0% (*w*/*v*) sucrose and 0.8% (*w*/*v*) agar [47]. The composition of the multipurpose solution was as follows (in mg·L^−1^): Ca(NO_3_)_2_·4H_2_O 467.6, KNO_3_ 232.3, KH_2_PO_4_ 272.0, K_2_SO_4_ 17.4, MgSO_4_·H_2_O 209.1, NH_4_NO_3_ 80.0, H_3_BO_3_ 1.4, NaMoO_4_·2H_2_O 0.12, MnSO_4_·4H_2_O 2.10, ZnSO_4_·7H_2_O 0.80, and CuSO_4_·5H_2_O 0.20. For Fe treatments, non-chelated iron sulfate (FeSO_4_), iron ethylenediaminetetraacetic acid (Fe-EDTA), or iron diethylenetriaminepentaacetic acid (Fe-DTPA) were added to the multipurpose medium with a final Fe concentration of 2.78 mg·L^−1^. The medium without any supplementary Fe was used as the control. The pH of the agar-solidified medium was adjusted to either 4.70, 5.70, or 6.70 using 1 M NaOH or HCl before autoclaving. Each treatment consisted of three replicates, and each replicate contained 12 shoots cultured in 3 containers.

### 4.3. Measurement of the Growth Characteristics

The data were collected after 6 weeks of culture, and the growth parameters such as the number of roots and leaves, the shoot, root, and stem length, leaf area, leaf color, the leaf fresh and dry weights, the relative water content and water content of leaves were measured. The fresh weight was measured with an electronic scale (EW 220-3NM, Kern and Sohn GmbH., Balingen, Germany). The saturated weight was measured after soaking the tissues in water by measuring the weight at every hour until the weight did not increase any more and the leaves are fully saturated. The dry weight was measured after drying the divided samples of the shoot and root for 72 h in a drying oven (Venticell-222, MMM Medcenter Einrichtungen GmbH., Munich, Germany) at 70 °C. The leaf area was measured with a leaf area meter (LI-3000, LI-COR Inc., Lincoln, NE, USA). The color values of leaves were measured with a color reader CR-11 (1994 Minolta Co., Ltd. Osaka, Japan). The relative water content and water content were determined using the following formulae [48,49]:

(1) $$\mathrm{Relative}\;\mathrm{water}\;\mathrm{content}=\frac{(\mathrm{fresh}\;\mathrm{weight}-\mathrm{dry}\;\mathrm{weight})\times 100}{\mathrm{saturated}\;\mathrm{weight}-\mathrm{dry}\;\mathrm{weight}}$$

(2) $$\mathrm{Water}\;\mathrm{content}\;=\frac{(\mathrm{fresh}\;\mathrm{weight}-\mathrm{dry}\;\mathrm{weight})\times 100}{\mathrm{fresh}\;\mathrm{weight}}$$

### 4.4. Chlorophyll Content

The contents of chlorophyll a and b were estimated according to Arono [50]. To determine the chlorophyll content, chlorophyll was extracted from 100 mg leaf tissues for 12 h with an 80% (*v*/*v*) acetone, and the absorbance was measured at 645 and 663 nm with a UV-spectrophotometer (Libra S22, Biochrom Ltd., Cambridge, UK). The contents of chlorophyll a and b were determined using the following formulae:

(3) $$\mathrm{Chlorophyll}\;a=\frac{\left(12.72\times \mathrm{OD}\;\mathrm{at}\;663\;\mathrm{nm}-2.59\times \mathrm{OD}\;\mathrm{at}\;645\;\mathrm{nm}\right)\times V}{\mathrm{Sample}\;\mathrm{fresh}\;\mathrm{weight}}$$

(4) $$\mathrm{Chlorophyll}\;b=\frac{(22.88\times \mathrm{OD}\;\mathrm{at}\;645\;\mathrm{nm}-4.67\times \mathrm{OD}\;\mathrm{at}\;663\;\mathrm{nm})\times V}{\mathrm{Sample}\;\mathrm{fresh}\;\mathrm{weight}}$$

(*V, the volume of the extraction solution. The chlorophyll content was expressed as mg of chlorophyll per g of fresh leaf weight).

### 4.5. Total Soluble Proteins and Antioxidant Enzyme Activities

100 mg leaf samples were homogenized in a 1.5 mL ice-cold 50 mM phosphate buffer (pH 7.0) containing 1 mM ethylenediaminetetraacetic acid (EDTA), 0.05% triton X-100, and 1 mM polyvinylpyrrolidone (PVP). The extracts were centrifuged at 13,000 rpm for 20 min at 4 °C, and the supernatant was used immediately for determination of the soluble protein contents and antioxidant enzyme activities. The soluble protein contents were measured with the Bradford Reagent (Sigma-Aldrich, St. Louis, MO, USA). The activities of superoxide dismutase (SOD), catalase (CAT), ascorbate peroxidase (APX), and peroxidase (POD) were measured according to the established protocols of Soundararajan et al. [51,52].

### 4.6. Activity of FCR

The composition of the reaction solution was as follows (in mol·L^−1^): 1 × 10^−6^ Fe (III)EDTA, 4 × 40^−4^ 2,2′-bipyridyl, 7.5 × 10^−4^ K_2_SO_4_, 6.5 × 10^−4^ MgSO_4_, 2.5 × 10^−4^ K_2_HPO_4_, 1 × 10^−3^ KCl, 1 × 10^−4^ H_3_BO_3_, 1 × 10^−6^ MnSO_4_, 5 × 10^−7^ CuSO_4_, 1 × 10^−6^ ZnSO_4_, 5 × 10^−8^ (NH_4_)_6_Mo_7_O_24_. Plants were placed with their roots in a 20 mL saturated CaSO_4_ solution for 5 min, and the roots were washed three times with distilled water, soaked for 20 min in the reaction solution (pH 5.3) in the dark. Then the absorbance was measured at 520 nm. The activity of FCR was determined using the following formula [53]:

(5) $$\mathrm{The}\;\mathrm{activity}\;\mathrm{of}\;\mathrm{FCR}=\frac{(\mathrm{OD}\;\mathrm{at}\;520\;\mathrm{nm})\times 10^{6}}{\mathrm{Root}\;\mathrm{fresh}\;\mathrm{weight}\times 8650}$$

### 4.7. Scanning Electron Microscopic (SEM) Analysis of Stomata

Leaf samples were cut into 0.5 mm^2^ pieces and fixed in 3.0% (*v*/*v*) glutaraldehyde (pH 7.5) at 4 °C overnight. Staining was done in a 1.0% osmium tetroxide solution for 2 h at 4 °C, then the samples were subsequently dehydrated in graded series of ethanol and were finally washed with 80% acetone. Dried samples were positioned on aluminum stubs with double stick tape prior to gold coating in a sputter coater (SC7640; Polaron, Sussex, UK). A field emission scanning electron microscope II (SEM/EDS, JSM-7610F, JEOL Ltd., Tokyo, Japan) was used to observe the stomata.

The size of open stomata and stomatal density were measured according to Jiyou Zhu [54]. Using multiscale segmentation and classification recognition as well as microscopy images of leaf stomata using a computer software (eCognition Development 64, Munich, Germany) with such, segmentation parameters as scale parameter 120, shape parameter 0.5, and compactness parameter 0.8.

### 4.8. Determination of Macro- and Micro-Nutrient Contents Using Inductively Coupled Plasma Spectrometer

The macro- and micro-nutrients were measured according to the method of Hailin Zhang [55]. Briefly, leaves dried in an oven at 60 °C was finely powdered, and then 500 mg samples were ashed in a Nabertherm muffle furnace (Model LV 5/11/B180, Lilienthal, Breman, Germany) at 525 °C for 4 h. The ash was dissolved in 5 mL 25% HCl, followed by dilution with 15 mL of warm distilled water and 10 mL room-temperature distilled water. The macro- and micro-nutrient contents were measured three times for each treatment using an inductively coupled plasma (ICP) spectrometer (Optima 4300DV/5300DV, Perkin Elmer, Germany).

### 4.9. Data Collection and Analysis

The statistical analysis was carried out using the Statistical Analysis Program (SAS 9.1, SAS Institute Inc., Cary, NC, USA). The experimental results were subjected to an analysis of variance (ANOVA) (*p* ≤ 0.05) and Duncan’s multiple range test (*p* ≤ 0.05). Graphing was performed with the OriginPro software (version 9.0).

## 5. Conclusions

Our results showed that the growth and development of *S. commixta* in vitro was affected by supplementary Fe. Fe-deficiency induced chlorosis, and decreased the biomass of plants. Application of supplementary Fe enhanced the number of leaves and roots, fresh/dry shoot and root weights, and the chlorophyll contents. Supplementary Fe also changed the macro- and micro-nutrient contents, especially Fe uptake in *S. commixta*. *S. commixta* had the best Fe absorption at pH 4.70. Among all the Fe sources and medium pH studied, Fe-EDTA at pH 5.70 was found to be the most effective in promoting the growth and development of *S. commixta* in vitro.

## Figures and Tables

**Table 1:** The growth parameters of *S. commixta* after 6 weeks of culture.

Fe Source (A)	pH (B)	Number of Leaves	Number of Roots	Shoot Length (cm)	Root Length (cm)	Stem Length (cm)	Stem Diameter (mm)	Leaf Area (cm^2^)	Fresh Weight (mg)		Dry Weight (mg)		Relative Water Content (%)	Water Content (%)
									Shoot	Root	Shoot	Root		
Control	4.70	4 b–d ^z^	1 cd	2.3 b–e	2.3 cd	1.7 a	2.24 ab	2.99 de	119.0 b–e	17.2 bc	33.6 bc	2.7 bc	63.70 a	75.51 a
	5.70	2 d	1 cd	2.3 b–e	2.2 cd	1.5 a	2.08 ab	2.60 de	157.7 b–d	14.9 bc	40.8 bc	1.3 c	79.36 a	67.14 a
	6.70	2 d	0 d	1.4 e	0.0 d	0.7 b	1.63 ab	1.88 e	55.4 e	0.0 c	22.0 c	0.0 c	63.94 a	64.73 a
FeSO_4_	4.70	6 ab	1 cd	3.1 a–c	2.8 cd	1.5 a	2.07 ab	5.79 b–e	182.1 bc	51.7 bc	47.9 ab	1.7 c	57.31 a	74.79 a
	5.70	7 a	4 ab	3.5 ab	10.6 a	1.4 a	2.34 a	8.74 ab	297.3 a	102.3 b	65.1 a	12.0 b	66.01 a	75.87 a
	6.70	5 a–c	2 b–d	3.1 a–c	6.7 a–c	1.7 a	1.74 ab	8.43 a–c	197.7 b	72.2 bc	32.6 bc	7.8 bc	92.28 a	77.31 a
Fe-EDTA	4.70	5 a–c	2 b–d	3.3 ab	6.3 a–c	1.5 a	1.99 ab	6.80 a–d	100.4 c–e	90.1 bc	27.5 bc	7.8 bc	55.81 a	61.81 a
	5.70	5 a–c	4 a	3.9 a	8.1 ab	1.3 a	2.28 a	10.54 a	296.1 a	225.5 a	71.6 a	19.4 a	65.34 a	75.58 a
	6.70	4 b–d	2 cd	3.0 a–d	2.9 cd	1.3 a	1.63 ab	5.00 b–e	120.0 b–e	42.7 bc	33.2 bc	4.5 bc	62.53 a	72.42 a
Fe-DTPA	4.70	2 d	0 d	1.5 de	0.0 d	1.5 a	1.19 b	2.22 e	70.9 de	0.0 c	24.5 bc	0.0 c	66.09 a	62.37 a
	5.70	3 dc	2 dc	1.7 c–e	0.9 d	1.5 a	1.80 ab	2.48 e	104.7 b–e	4.5 bc	37.2 bc	1.0 c	73.42 a	60.62 a
	6.70	4 b–d	3 a–c	2.6 a–e	4.5 b–d	1.4 a	1.74 ab	4.40 c–e	103.7 b–e	49.0 bc	31.1 bc	5.3 bc	56.50 a	73.99 a
F-test ^y^	A	***	**	***	***	NS	NS	***	***	***	*	***	NS	NS
	B	**	***	NS	NS	NS	NS	NS	***	NS	**	NS	NS	NS
	A × B	***	***	NS	**	NS	NS	NS	*	**	*	**	NS	NS

^z^ Mean separation within columns for each cultivar by Duncan’s multiple range test, where different letters indicate the significant differences at *p* = 0.05. ^y^ NS, *, **, ***, Not significant or significant at *p* = 0.05, 0.01, or 0.001, respectively.

**Table 2:** The leaf color of *S. commixta* after 6 weeks of culture.

Fe Source (A)	pH (B)	L*	a*	b*
Control	4.70	72 b ^z^	−21 d	51 bc
	5.70	50 c	−14 c	28 fg
	6.70	22 fg	−24 a	27 g
FeSO_4_	4.70	35 e	−31 f	34 de
	5.70	27 fg	−27 ef	33 ef
	6.70	80 a	−14 c	60 a
Fe-EDTA	4.70	39 de	−25 e	39 d
	5.70	42 de	−12 c	25 gh
	6.70	73 ab	−13 c	52 b
Fe-DTPA	4.70	19 g	9 b	14 i
	5.70	23 fg	12 b	20 h
	6.70	46 cd	−27 ef	46 c
F-test ^y^	A	***	***	***
	B	***	***	***
	A × B	***	***	***

L*, lightness; a*, red (+)/green (−) color attribute; b*, yellow (+)/blue (−) color attribute values. ^z^ Mean separation within columns for each cultivar by Duncan’s multiple range test, where different letters indicate the significant difference at *p* = 0.05. ^y^ ***, Significant at *p* = 0.001.

**Table 3:** Mineral contents in *S. commixta* after 6 weeks of culture (mg·g^−1^ dry weight).

Fe Source (A)	pH (B)	K	Ca	Mg	Zn	Mn	Fe	Cu	B	Si	P	S
Control	4.70	279.27 a ^z^	132.20 b	41.59 b	1.16 b	1.08 b	1.75 i	0.29 a	0.32 e	1.16 e	117.53 b	28.99 c
	5.70	212.03 d	120.27 c	31.17 f	1.03 c	0.68 f	1.78 i	0.23 b	0.37 b	1.62 c	102.43 c	23.24 f
	6.70	159.40 h	100.00 f	27.57 i	0.98 e	0.52 h	1.55 j	0.18 cd	0.34 d	1.00 f	92.43 e	18.03 h
FeSO_4_	4.70	252.00 b	113.33 d	32.47 e	1.01 d	0.89 d	4.44 e	0.28 a	0.35 cd	1.43 d	99.98 cd	25.96 d
	5.70	234.47 c	94.24 g	27.72 i	1.37 a	1.35 a	3.54 f	0.17 e	0.61 a	2.61 a	77.56 gh	30.36 b
	6.70	189.40 f	87.51 h	29.69 g	0.63 k	0.58 g	2.06 h	0.17 e	0.26 h	2.55 b	76.66 h	18.59 h
Fe-EDTA	4.70	236.93 c	99.46 f	34.84 d	0.82 g	0.83 e	6.62 d	0.19 c	0.27 g	0.91 g	101.70 c	24.88 e
	5.70	278.60 a	145.27 a	46.27 a	0.89 f	1.02 c	3.00 g	0.17 e	0.37 b	0.77 i	125.10 a	31.56 a
	6.70	214.20 d	130.20 b	36.78 c	0.71 i	1.10 b	1.86 i	0.13 f	0.35 c	0.82 h	101.13 c	31.51 a
Fe-DTPA	4.70	176.97 g	77.17 j	20.41 k	0.62 k	0.54 h	18.61 a	0.18 de	0.19 i	0.69 j	79.62 g	21.11 g
	5.70	202.47 e	104.77 e	28.56 h	0.67 j	0.69 f	13.69 b	0.13 f	0.30 f	0.56 k	88.72 f	25.06 e
	6.70	186.27 f	83.03 i	24.21 j	0.80 h	0.52 h	9.80 c	0.10 g	0.25 h	0.92 g	97.73 d	18.19 h
F-test ^y^	A	***	***	***	***	***	***	***	***	***	***	***
	B	***	***	***	***	***	***	***	***	***	***	***
	A × B	***	***	***	***	***	***	***	***	***	***	***

^z^ Mean separation within columns for each cultivar by the Duncan’s multiple range test, where different letters indicate the significant difference at *p* = 0.05. ^y^ ***, Significant at *p* = 0.001.

## Data Availability

*Sorbus commixta* plants used in this study were kindly provided by Prof. Byoung Ryong Jeong, Gyeongsang National University, Jinju 52828, Korea.
